# Gut Bacteria and their Metabolites: Which One Is the Defendant for Colorectal Cancer?

**DOI:** 10.3390/microorganisms7110561

**Published:** 2019-11-13

**Authors:** Samira Tarashi, Seyed Davar Siadat, Sara Ahmadi Badi, Mohammadreza Zali, Roberto Biassoni, Mirco Ponzoni, Arfa Moshiri

**Affiliations:** 1 Microbiology Research Center, Pasteur Institute of Iran, 1316943551 Tehran, Iran; tarashisamira@gmail.com (S.T.); d.siadat@gmail.com (S.D.S.); sarahmadi@gmail.com (S.A.B.); 2 Mycobacteriology and Pulmonary Research Department, Pasteur Institute of Iran, 1316943551 Tehran, Iran; 3 Gastroenterology and Liver Diseases Research Center, Research Institute for Gastroenterology and Liver Diseases, Shahid Beheshti University of Medical Sciences, 19857-17411 Tehran, Iran; nnzali@hotmail.com; 4 Laboratory of Molecular Medicine, IRCCS Instituto Giannina Gaslini, 16147 Genova, Italy; Robertobiassoni@gaslini.org; 5 Laboratory of Experimental Therapies in Oncology, IRCCS Istituto Giannina Gaslini, 16147 Genova, Italy

**Keywords:** colorectal cancer, gut bacteria, dysbiosis, epigenetics

## Abstract

Colorectal cancer (CRC) is a worldwide health concern which requires efficient therapeutic strategies. The mechanisms underlying CRC remain an essential subject of investigations in the cancer biology field. The evaluation of human microbiota can be critical in this regard, since the disruption of the normal community of gut bacteria is an important issue in the development of CRC. However, several studies have already evaluated the different aspects of the association between microbiota and CRC. The current study aimed at reviewing and summarizing most of the studies on the modifications of gut bacteria detected in stool and tissue samples of CRC cases. In addition, the importance of metabolites derived from gut bacteria, their relationship with the microbiota, and epigenetic modifications have been evaluated.

## 1. Introduction

Colorectal cancer (CRC) remains a significant troublesome health issue worldwide [1]. This multifactorial and widespread cancer counts as one of the most common causes of cancer-related death [2]. CRC has a close association with lifestyle, clinically affecting the large intestine and rectum [3,4]. Recent evidence suggests that dysregulation of microbiota-host interactions is associated with various diseases, including diabetes, bowel disease, and cancer [5,6]. In addition to genetic and environmental factors, such as inflammatory processes, diet, alcohol consumption, and smoking, dysbiosis of gut bacteria and epigenetic modifications are a critical link to an increased risk of CRC [7,8]. The term “dysbiosis” refers to an imbalance in the community of healthy human microbiota [9], known as the microbial community inhabiting the skin, oral cavity, lower respiratory tract, vagina, urinary tract and gut [10]. The highest and most varied bacterial density are inhabitants in the large human bowel and interact with the host in a symbiotic relationship [11]. Recent evidence links the response to anticancer immune checkpoint inhibitor therapy to the presence of specific species in the microbiota of patients [12,13,14]. In addition, microbial-derived metabolites also play a fundamental role in host metabolism and CRC progression [15,16]. Therefore, a growing interest in the determination of a possible link between gut bacteria and CRC has been aroused in the last few decades [17,18]. However, causative genera in CRC evolution remains poorly defined. Nevertheless, there is a gap in knowledge on the role of various gut bacteria and their metabolites in CRC, also considering that epigenetic modifications play a significant role in CRC development. Thus, the complete mechanism at the base of CRC pathogenesis is not fully understood and the different aspects of bacterial effects are also entirely unclear. Currently, a major challenge is to define how to integrate microbiota data into medicine approaches in order to introduce an effectiveness prevention, diagnosis, and treatment strategies. The current study furthermore provides a detailed overview of the most critical gut bacteria DNA detected in the standard sample types of CRC. Finally, the most important mechanisms, microbial-derived metabolites, and epigenetic modifications that influence progression to CRC are discussed briefly.

## 2. The Intestinal Bacteria in Times of CRC:

**Figure 1:**
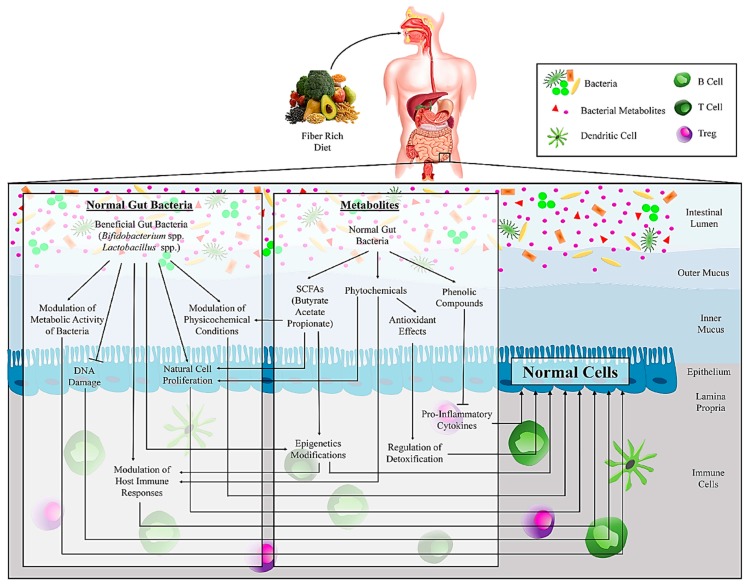
The schematic association of the gut bacteria and their metabolites in maintaining cell homeostasis.

**Figure 2:**
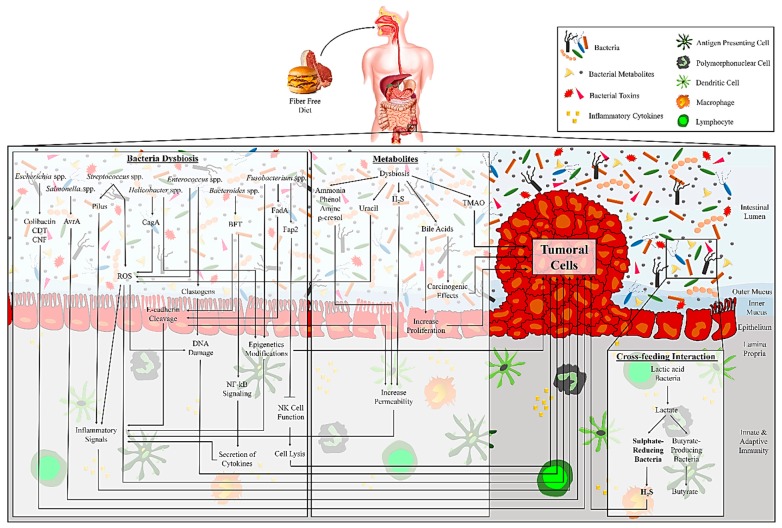
The schematic association of gut bacteria and their metabolites, which affects the development of tumorigenesis.

Humans are known as “superorganisms” because of their inherent ability to organize the microbial communities in addition to their cells [19]. The gut bacterial population consists of different phyla species [20]. These organisms have a significant effect on several essential aspects of human health, including nutrient absorption, physiology, metabolism, immune function, and protection against pathogens. Valuable insights have been recently gained into the dysbiosis of the gut bacteria in the development of CRC [19,21]. Some of the most important insights of gut bacteria affects this development, which is discussed in the following sections and is summarized in Figure 1 and Figure 2. There is a long history of association between gut bacteria and CRC progression, which was first introduced by Reddy et al. in 1975 [22], suggesting that a bacteria-dependent dysregulation in the immune system can alter the host metabolism. However, how microbiota can influence CRC development has been a topic of great discussion. A recent study proposed a model that highlights the role of some bacteria as drivers or passengers [23], indicating that pathogenic bacteria (driver) at first rapidly colonize the intestinal epithelium, while opportunistic microorganisms (passengers) then enrich the cancer condition. Accordingly, bacteria with pro-carcinogenic capabilities, especially opportunistic pathogens and polymicrobial anaerobic bacteria, are often detected in early CRC stages [24]. Indeed, a high proportion of bacteria belonging to the *Shigella*, *Salmonella*, and *Citrobacter* genera have been found in early CRC stages compared to healthy controls, while they vanished in a more advanced stage of CRC development [21]. In contrast, the presence of *Fusobacterium* ssp. and *Streptococcaceae* families, as passenger bacteria, has not been found in early CRC stages. While, at first the passenger bacteria may use the benefits of changes in the tumor microenvironments to thrive better and expand [25,26], their high proportion in the first stages of CRC might have a role in cancer development [21]. Nakatsu et al. examined bacterial changes across the CRC stages [27]. The enrichment of *Fusobacterium*, *Gemella*, *Leptotrichia,* and *Parvimonas* and the losses of *Alistipes*, *Bacterioides*, *Blautia*, *Collinsella* have been reported in early stages (Stage I-II) CRC. Neither of these variations was detected significantly in late stages (Stage III-IV) CRC. Zeller et al. reported a strong enrichment of *Fusobacterium* and *Peptostreptococcus* and losses of *Eubacterium* and *Streptococcus* in the early stages of CRC [28].

## 3. The Importance of Gut Bacteria Detected in the Stool and Tissue in CRC:

Several studies compare the evaluation of microbiota derived from tissue and stool samples of CRC patients and healthy controls. As an example of guidance, the data on significant relative abundance of gut bacterial genera in CRC cases are presented in Figure 1, Figure 2, and Table 1. In addition, several studies (not shown in Table 1) evaluated the variation of gut bacteria between tumor tissue and its healthy adjacent tissue in CRC patients [24,26,29,30,31,32,33,34,35]. The incidence risk of CRC is higher in developed countries than in developing ones, which is highly related to dietary differences. However, most of the studies evaluated gut bacteria and CRC in developed countries except for some studies from Malaysia, Indonesia, India, and Morocco [36,37,38,39]. Evidence based on global epidemiological studies suggests an increased risk of CRC by high caloric intake and consumption of some diets like protein (red meat) and animal fat and low consumption of multivitamins and fibers, which affects gut microbial metabolism [40,41]. In the case of local CRC, the range of cure effectiveness is from 70%–90%, while a high mortality rate is reported in advanced CRC cases [42]. Overall, the worldwide incidence of CRC is approximate 4%–5%, and personal traits and lifestyle are considered the most significant risk factors [43]. Moreover, a significant role for CRC development has been ascertained for the dominant gut bacteria [43], although, it is currently unclear how dysbiosis could progress CRC.

Herein, some crucial gut bacterial mechanisms involved in CRC are discussed in detail. Based on molecular methods, one of the first studies identified a secure link between the genus of *Escherichia* and CRC [42]. Indeed, *Escherichia*, a commensal gut microbiota, increased in the colon of CRC patients compared with healthy individuals, and some strains, like phylogroups B2 and D, are frequently linked to CRC [44]. The genotoxin colibactin, produced by the polyketide synthase genomic island, *pks*, presents in *E. coli* strains of the phylogenetic group B2 and can contribute to the development of CRC [45]. Other *E. coli* strains that are closely related to CRC can produce a cytotoxic necrotizing factor (CNF) or cytolethal distending toxin (CDT) [46]. CRC and *Streptococcus* bacteremia have also shown a close association since 1951, when a case of enterococcal endocarditis from *S. bovis* in association with CRC was reported [47]. Approximately 25%–80% of cases with *S. bovis* bacteremia progress to CRC, but the primary mechanisms are not identified [47,48]. However, *S. bovis* and its antigen can stimulate the production of IL-8 in the colon [49] that in turn might contribute to colon carcinogenesis by the induction of NO and ROS [47]. Also, *S. gallolyticus* subspecies *gallolyticus*, as biotype 1 *S. bovis*, has shown a strong association with CRC [50,51]. This organism has been detected in 20%–50% of CRC and colorectal adenoma (CRA) cases [52], the latter being known as a noncancerous colon tumor, which may progress into CRC. *S. gallolyticus* encodes a pilus with a collagen-binding domain that is more advantageous for CRC development [52,53], through inflammatory signals produced by its pilus [53,54].

In the genera of *Bacteroides*, *B. fragilis* strains comprise approximately 0.1% of healthy gut microbiota. The *B. fragilis* toxin (BFT) of enterotoxigenic *B. fragilis* (ETBF) has been linked to CRC [55,56], since it was found in 38% of isolates from CRC cases compared with 12% of healthy controls [57]. BFT induces the cleavage of E-cadherin and enhances CRC proliferation and expression of *Myc* as a proto-oncogene. In addition, BFT initiates NF-kB signaling and induces secretion of cytokines that finally lead to the contribution of mucosal inflammation [42,58]. Another suspected bacterial genus among CRC subjects is *Enterococcus*. Some *E. faecalis* strains can stimulate the production of ROS and superoxide anions and to induce genomic instability by DNA damaging [42]. *E. faecalis* can induce the production of diffusible clastogens, a chromosomal-breaking factor, that causes DNA damages [59]. Therefore, these strains have been proposed as motivators and boosters of CRC. Moreover, the genus *Fusobacterium* appears as a dominant phylotype influencing CRC. This conclusion is supported by the association between the abundance of *Fusobacterium* and NF-kB-driven inflammatory genes in human CRCs [42]. Specifically, the abundance of *F. nucleatum* in CRC is correlated with high production of pro-inflammatory cytokines, leading to upregulation of NF-kB [60]. The carcinogenic properties of *F. nucleatum* strains are mediated by the unique adhesin, FadA (FadAc) [61], through binding to E-cadherin, with consequent activation of cell growth-related signaling pathways [42]. Moreover, *F. nucleatum* can inhibit tumor cell lysis by an interaction between its Fap2 protein and NK cells receptors, thus inhibiting the cytotoxic potential of NK cells [62]. Furthermore, *Salmonella* might enhance CRC-risk through activation of signaling pathways by its pathogenic product, AvrA [63]. On the other hand, the role of *Helicobacter pylori* in CRC remains controversial, although some new research has introduced the role of *H. pylori* cytotoxin-associated gene A (CagA), as well as the production of ROS and NOS, in the induction of inflammatory pathways and CRC progression [64,65]. Some meta-analysis studies have also reported a high risk of CRC in *H. pylori* positive patients, especially in the early stage of CRC [66,67]. Finally, *Clostridium septicum* infection has been clinically linked to CRC [68], but the related underlying mechanism remains indefinite, and no direct association has been identified. It has been only suggested that *C. septicum* spores can easily germinate in the hypoxic and acidic tumor condition [69].

Although the above examples indicated the adverse effects of gut bacteria on CRC progression, some positive impacts on CRC prevention have been similarly detected. Frequently, the mechanisms of potentially probiotic gut bacteria are investigated in animal models [70,71]. Nevertheless, several human clinical trials have taken into consideration the protective effect of different probiotics in CRC patients [72,73]. The term “probiotic” refers to the prescription of some live bacteria which provide health benefits [74]. For instance, *Bifidobacterium longum* and *Lactobacillus acidophilus* have been introduced as inhibitors of CRC progression [75,76]. *L. acidophilus* seems to influence the 1,2-dimethylhydrazine-induced CRC, used as a carcinogen agent in the gut lumen, and to reduce the risk of CRC progression in rats [75]. *B. adolescentis* and *B. infantis* also suppress 3-methylcholanthrene-induced CRC in the mice model [77]. Also, the protective effect of *L. acidophilus* in CRC patients seems to derive from its binding to carcinogens in the human gut lumen, thus decreasing intestinal cell proliferation [78]. A clinical trial has indicated the effect of *L. casei* on the reduction of CRC recurrence [79], while other studies point to the protective effect of *L. rhamnosus* GG and *B. lactis* Bb12 in CRC patients [70,80]. Generally, the protective effects of the beneficial gut bacteria in CRC cases is mostly due to the reduction of DNA damage, intestinal cell proliferation, and secretion of interleukin-2, and to the enhancement of host immune responses, interferon-γ production, and modification of physicochemical conditions and metabolic activity of bacteria in the gut [70,80,81]. The increases and decreases of gut bacteria reported in the different studies analyzed are highlighted in Table 1. In addition, Table 1 depicts the use of different techniques of analysis as one of the most important reasons for the vast variations observed. Until the end of the last century, the association between gut bacteria and CRC were identified by culture methods [82,83]. Therefore, the vast majority of gut bacteria that have been recently associated with CRC remain uncharacterized due to their unfeasibility of culturing. The development of molecular techniques, mainly based on the analysis of hypervariable region of 16S ribosomal RNA (rRNA) gene, has provided a large amount of data and lead to better characterization of various bacterial communities [19,47]. Indeed, high throughput sequencing techniques have vastly expanded our knowledge of the significant role of gut bacteria in CRC development [84].

## 4. Microbial-derived Metabolites and CRC:

New aspects are quickly coming to the fore as possible players of gut bacteria in CRC progression. Different types of diet potentially control the production of microbial-derived metabolites, which have an essential influence on host metabolism and CRC development (Figure 1 and Figure 2) [21]. The data on significant microbial-derived metabolites in stool samples of CRC cases are presented in Table 2. Data are based on The Human Metabolome Database (http://www.hmdb.ca/). The status of all of the reported microbial-derived metabolites in stool samples of CRC cases was “detected but not quantified”. In general, consumption of dietary fiber, which is neither digested nor absorbed, is known as one of the effective strategies to modulate the gut bacteria composition, even for the ones introduced as being potentially prebiotic [144]. The term “prebiotic” refers to selectively food products that induce specific beneficial changes in the gut bacterial community of the host [145]. The association between fiber consumption and gut bacterial pattern is highly under the influence of type of consumed fiber. Different classifications, including origin, physicochemical characteristics, chemical composition, and other subclassifications based on carbohydrate chain length are introduced to describe dietary fibers, because of their heterogeneous nature. The Codex Alimentarius Commission is classified dietary fibers as edible naturally carbohydrates in consumed foods, edible manipulated carbohydrates by enzymatic, chemical or physical modifications in food row materials and edible synthetic carbohydrates [144]. All of them established beneficial physiological effects which approved by scientific evidence and can impact fermentation of different types of gut bacteria and therefore, therapeutic effects of consumers. With regard to physicochemical characteristics, dietary fibers can be separated based on solubility, fermentability, and viscosity. Solubility is indicated to highly impact on the fermentation caused by gut bacteria [144]. Soluble fiber, e.g., pectin and gums, easily digest in the proximal colon and mostly as part of the body metabolism caused by the reduction of carbohydrate absorption, blood pressure, insulin, and LDL level [146]. While insoluble fiber, e.g., cellulose and lignin, is partially fermented in the distal colon, the bacterial density is higher and is commonly involved in intestinal health. In general, fiber from vegetables and fruit is mainly soluble, and cereal fiber is mostly insoluble [147]. The gut bacteria begins fermentation of undigested dietary fibers in the large intestine and produces a huge variety of metabolites [148]. The most original products of gut bacteria in the colon during the fermentation process are short-chain fatty acids (SCFAs), like butyrate, acetate, and propionate, which are modulated by a fiber-rich diet [149]. Butyrate and propionate influence on the regulation of gut physiology and immune system, while acetate is a substrate in gluconeogenesis and lipogenesis process [145]. The members of the phylum *Firmicutes* frequently produce butyrate, which induces several controversial actions in the colon [150]. There are plenty of data describing the role of butyrate in cancer prevention, but its role in CRC remains inconclusive. Butyrate stimulates the natural proliferation of epithelial cells in the colon [151]. In addition, the phenolic compounds, by inhibiting several pro-inflammatory mediators, can lead to alterations of the gut bacterial community [152]. Nevertheless, its capability to interact dependently on the genetic backgrounds has increased concerns about its role in CRC development [21]. Consequently, considering the type of microbial-derived metabolites is essential, but their interaction with genetic and epigenetic backgrounds are challenging tasks that also need to be considered.

Despite the beneficial SCFAs fermentation, amino acids can produce potentially harmful compounds during fermentation. Some of these, like ammonia, p-cresol, hydrogen sulfide, and some amines, may be important in CRC and in other gut disorders, which is controlled by a fiber-free diet [115,153]. These compounds may increase the risk of DNA damage, leaky gut, inflammation, and CRC development [153]. For instance, a secure connection has been established between gut bacteria and the metabolism of sulfate to produce cysteine, methionine, and hydrogen sulfide (H_2_S) that, in turn are toxic in high concentrations and contribute to the proliferation of colon cells and CRC progression [154]. Production of H_2_S in the gut is mostly done by members of *Desulfovibrio* spp., as specialist sulfate-reducing bacteria. They can utilize lactate to improve their growth, and sulfide formation [155] to stimulate CRC progression by the inhibition of butyrate oxidation and by inducing the breakdown of the gut barrier. The level of hydrogen sulfide is mainly influenced by bacterial activity, rather than by their abundance [156,157]. Butyrate-producing bacteria can also utilize lactate in competition with sulfate-reducing bacteria, especially *Desulfovibrio* spp. Lactate is one of the beneficial products of colonized lactic acid gut bacteria, including *Lactobacillus*, *Streptococcus*, *Bifidobacterium*, *Enterococcus*, and *Eubacterium*, which usually utilized by other gut bacterial genera in a cross-feeding interaction [158]. An evaluation is compared produced butyrate of *Eubacterium hallii* and *Anaerostipes caccae*, as two main butyrate-producing bacteria, from lactate in coculture with *Desulfovibrio piger* [155]. The results confirmed the high reduction of produced butyrate from lactate in this condition. In addition, the results of the triculture experiment involving *Bifidobacterium adolescentis*, as a lactic acid gut bacteria, have been strongly established inhibition of butyrate formation and induction of sulfide formation in the presence of *Eubacterium hallii*, *Anaerostipes caccae* and *Desulfovibrio piger*. Similarly, a high level of amines, especially polyamines, are toxic and are associated with CRC [157]. Several gut bacteria like *Salmonella enterica* subsp. *enterica* serovar Typhimurium, *S. flexneri*, *H. pylori,* and *S. pneumonia*, increase their virulence by abuse of polyamines [159]. Phytochemicals are also crucial because of their antioxidant effects and their potency in the regulation of detoxification, cell proliferation, apoptosis, and inflammation [160]. The reactive oxygen species (ROS), can damage DNA and increase the risk of CRC through neutralizing the antioxidants [157]. The nitrogen metabolites, like N‑nitroso compounds (NOCs), potentially promote CRC by the induction of DNA damage [157].

It has been postulated that an imbalance in the gut bacterial community can enhance the proliferation of damaging bacteria and their carcinogenic products [161]. However, additional investigations are required to establish this hypothesis. Bile acids can induce cytotoxic effects and increase the proliferation of malignant cells [162]. Overall, bile acids, like deoxycholic acid and lithocholic acid, have been potentially introduced as carcinogenic agents having a negative correlation with the level of anti-carcinogenic products in the colon [163]. Uracil, another microbial-derived metabolite, is also associated with ROS production in the intestine [164]. Gut bacteria metabolism can also induce trimethylamine N-oxide (TMAO), which is intensely associated with CRC [165]. Furthermore, many gut bacteria, via ethanol induction, produce highly carcinogenic acetaldehyde [166]. Generally, the fermentation is not the only metabolism process of gut bacteria; indeed they can also induce anaerobic metabolism. For example, sulfate, nitrate, and different organic compounds can function as electron receptors in the respiratory process [167]. Also, oxygen may count as an electron receptor of the facultative anaerobes *Bacteroides* spp. and *Faecalibacterium prausnitzii* [168,169].

In addition to the direct effect of gut bacteria and their metabolites on the development of homeostasis and tumorigenesis, they can be indirectly involved. For instance, bacteria commonly exchange primary metabolites with other organisms, known as cross-feeding interaction [170]. Dietary fiber extensively increases metabolic interaction in the gut bacterial community [144]. Competition of sulfate-reducing bacteria and butyrate-producing bacteria on exchanging of produced lactate by lactic acid bacteria in order to produce H2S or butyrate in different conditions is one of the most identified cross-feeding examples [155]. In addition, some gut bacteria utilize hydrogen and formate, and they mainly participate in anaerobic metabolism through a cross-feeding interaction [156]. These interactions play a vital role in the formation of gut microbial communities [170]. In brief, it can be concluded which a complex bidirectional network involved in the regulation of gut bacterial community by metabolites and metabolites by the gut bacterial community.

## 5. The Role of Bacterial Metabolites in Epigenetic Modifications of CRC

It is well known that epigenetic modifications influence many cellular processes by regulating gene expression, notably without direct modification of DNA sequence in the genome. Several types of epigenetic modifications, including histone modifications, DNA methylation, chromatin remodeling, and RNA-based regulation, are identified [172]. However, the value of epigenetic modifications in the development of different disorders in comparison with genetic mutations had been mostly ignored. With the increasing knowledge of the potential association between epigenetics and gene expression, evaluation of epigenetic modifications in different disorders has become a popular area of research [7]. Bacteria and their metabolites have a profound effect on the transcriptional profile of the host cells by the induction of epigenetic modifications [177]. These metabolites are crucial messengers in the crosstalk between microbiota and host cells, and microbiota can cooperate in the development of several major disorders by induction of epigenetic modifications [7]. A growing area of interest is the association between different epigenetic modifications in CRC progression and gut bacteria. Epigenetic regulation of many common genes (like *GATA4*, *MLH1*, *p16INK4a*, *LKB1*, and *APC*) and genetic pathways in CRC are well documented [178]. As mentioned, SCFAs are known as the major products of gut bacteria, which induce histone modification [179]. Butyrate and acetate act as histone deacetylase inhibitors, which affect the epigenetic modifications governing CRC development [180]. Propionate is known as a less effective histone deacetylase inhibitor, with respect to butyrate, because of its higher bioavailability and lower accumulation in colonocytes [178]. In particular, *Faecalibacterium*, *Eubacterium*, and *Roseburia* were identified as the most important butyrate producer in the gut microbiota. However, other butyrate-producers also have been found, such as *Fusobacterium*, *Peptoniphilus*, *Coprococcus*, *Porphyromonas*, *Clostridium*, *Megasphaera,* and others [181]. Evidence indicates that *Fusobacterium* increases methylation of the *hMLH1* gene and microsatellite instability [182]. The loss of histone H4 lysine monoacetylation and H4K16 and H4K20 trimethylation has been identified as a hallmark in CRC [183]. A detailed evaluation indicated acetylation of H3K27 along with methylation of H3K4 as the possible cause of activation of variant enhancer loci in tissue samples of CRC cases [184]. Besides, trimethylation of H3K4, H3K9, and H4K20 has been also evaluated in CRC [185]. Also, gut bacteria produce methionine during the metabolism of sulfate. Methionine modulates bacterial metabolism to increase S-adenosyl methionine (SAM) synthesis, which is a methyl donor for DNA methyltransferase [186]. *F. nucleatum* was also found concerning DNA methylation by targeting innate immune signaling [187]. Several investigations of CRC epigenome have introduced numerous aberrant methylated genes in CRC cases, such as *RAAS F2A*, *WIF1*, *ALX4*, *MGM2*, *APC*, *RUNX3*, *p14*, *p16*, *SOX2*, and *NDRG4* [188,189,190]. It is noteworthy that aberrant methylation of *cMyc* gene, encoding the c-myc oncoprotein, has been detected in CRC cases [191]. Moreover, *H. pylori* induces methylation of some genes related to cell growth, cell adherence, and DNA repair [192]. Besides, trimethylamine, mainly produced by *Escherichia coli,* induces DNA methylation [179]. Also, dysregulation of miRNAs, potential cancer biomarkers, is frequently reported in many studies [193,194]. For instance, overexpression of miR-21 and miR-106 has been detected in stool samples of CRC cases [195] and *F. nucleatum* has been shown to decrease the miR-18a level and to modulate some innate immune signaling in CRC [196]. In addition, an array of candidate miRNAs, which are involved in different process like signaling, proliferation, apoptosis, differentiation, migration, and invasion (*i.e.,* let-7 family, miR-17–92, miR-34a, miR-34b/c, miR-92a, miR-135a/b, miR-139, miR-145, miR-126, miR-133b, miR-141, miR-143, miR-144, miR-192, miR-195, miR-200c, miR-215, and miR-675) have been suggested in association with CRC [195,196,197,198]. Overall, various links have been found between different miRNAs and gut bacteria to impact on CRC developments [199]. In summary, several studies have explained in more detail the crosstalk between the microbiota and epigenetic modifications in CRC [7,178,198]. It is suggested that the prescription of *L. acidophilus*, *L. casei*, and *B. breve* in CRC cases can enhance expression of some tumor suppressor genes, which were typically suppressed by methylation process [180]. To date, the existing data about the epigenome strongly validate the fact that epigenetic factors rather than genetics could account as more precise disease pathogenetic biomarkers. In this context, further studies are required to deeply explore the correlation between epigenetic modifications and microbiota in CRC subjects.

## 6. Conclusions

Emerging scientific advances of the role of gut bacteria community in the pathogenesis of CRC continue to be elucidated and refined. Given existing evidence of dysbiosis in CRC, the link between gut bacteria and CRC development has become an urgent topic for future biomedical research. We tried to review the effect of the gut bacteria community and their metabolites in CRC cases and the salient epigenetic mechanisms. Ultimately, the combined use of epigenetic, microbiota, and metabolites analyses can be very significant for reaching a targeted therapeutics and innovative precision strategy for CRC. Therefore, introducing a personalized modulation of the pattern of gut bacteria and their metabolites activity or epigenetic modifications may be a new and useful approach to reduce the risk of CRC progression.

## Figures and Tables

**Table 1:** Evidence of relative abundance of gut bacterial genera isolated from stool and tissue samples of CRC patients.

Gut bacteria	Author	Published Time	Enrolment Time	Country	Sample Type (S/T^a^)	Cancer Type	Method
**Increased Gut Bacteria**							
*Acidaminobacter*	Weir [85]	2013	-	USA	S	CRC	16S rDNA Sequencing
*Acidovorax*	Sanapareddy [86]	2012	-	USA	**T**	CRA	16S rDNA Sequencing
*Actinomyces*	Peters [87]	2016	2012–2014	USA	S	CRC/CRA	Pyrosequencing
	Kasai [88]	2015	2012–2013	Japan	S	CRC/CRA	T-RFLP/NGS
*Akkermansia*	Weir [85]	2013	-	USA	S	CRC	16S rDNA Sequencing
	Mira-Pascual [89]	2015	-	Spain	T	CRC/CRA	qPCR
*Alistipes*	Feng [90]	2015	2010–2012	Austria	**S**	A-CRA^b^/CRC	Metagenomic Shotgun Sequencing
	Wu [91]	2013	-	China	**S**	CRC	16S rRNA Sequencing
*Atopobium*	Vogtmann [92]	2016	1985–1987	USA	**S**	CRC	Whole-genome Shotgun Sequencing
	Kasai [88]	2015	2012–2013	Japan	**S**	CRC/CRA	T-RFLP/NGS
	Ahn [93]	2013	1985–1989	USA	**S**	CRC	16S rDNA Sequencing
*Anaerococcus*	Chen [94]	2012	-	China	T	CRC	16S rDNA Sequencing
*Anaerotruncus*	Chen [94]	2012	-	China	T	CRC	16S rDNA Sequencing
*Aquabacterium*	Sanapareddy [86]	2012	-	USA	T	CRA	16S rDNA Sequencing
*Bacteriodes*	Feng [90]	2015	2010–2012	Austria	**S**	A-CRA/CRC	Metagenomic Shotgun Sequencing
	Sobhani [95]	2011	2004–2006	France	**S**	CRC	Pyrosequencing/qPCR
	Kasai [88]	2015	2012–2013	Japan	**S**	CRC/CRA	T-RFLP/NGS
	Xu [96]	2017	-	China	**T**	CRC/CRA	NGS
	Brim [97]	2013	-	USA	**S**	Colon polyps	16S rRNA Sequencing/HITChip/Pyrosequencing
	Flemer [98]	2015	-	Ireland	**S/T**	CRC/Polyps	16S rDNA Sequencing
	Wang [99]	2012	-	China	**S**	CRC	16S rDNA Sequencing
	Wu [91]	2013	-	China	**S**	CRC	16S rDNA Sequencing
	Chen [94]	2012	-	China	**T**	CRC	16S rDNA Sequencing
	Drewes [37]	2017	-	Malaysia	**T**	CRC	16S rDNA Sequencing
	Gao [100]	2015	-	China	**T**	CRC	16S rDNA Sequencing
	Liang [101]	2016	-	China	**S**	CRC	duplex qPCR
	Nakatsu [27]	2015	2011–2014	China	**T**	CRC/CRA	qPCR
	Huipeng [102]	2014	-	China	**T**	Colon cancer	PCR/DGGE
*Bifidobacterium*	Nugent [103]	2014	-	USA	**T**	CRA	qPCR
*Bilophila*	Feng [90]	2015	2010–2012	Austria	S	A-CRA/CRC	Metagenomic Shotgun Sequencing
	Hale [104]	2017	2001–2005	USA	S	CRA	16S rDNA Sequencing
	Hibberd [105]	2017	-	USA	T	Colon cancer	16S rDNA Sequencing
	Yazici [106]	2017	2011–2012	USA	T	CRC	16S rDNA Sequencing
*Blautia*	Ai [107]	2017	2012	China	S	CRC/CRA	16S rRNA Sequencing
	Wu [91]	2013	-	China	S	CRC	16S rRNA Sequencing
	Mira-Pascual [89]	2015	-	Spain	**T**	CRC/CRA	16S rRNA Sequencing/qPCR
*Butyrivibrio*	Dejea [108]	2014	-	USA	T	CRC/CRA	Pyrosequencing
*Campylobacter*	Xu [96]	2017	-	China	T	CRC/CRA	NGS
	Wu [91]	2013	-	China	S	CRC	16S rRNA Sequencing
	Zhang [109]	2018	2014–2015	China	S	CRC/A-CRA/BP ^c^	16S rRNA Sequencing
*Citrobacter*	Weir [85]	2013	-	USA	S	CRC	16S rDNA Sequencing
*Cloacibacterium*	Sanapareddy [86]	2012	-	USA	T	CRA	16S rDNA Sequencing
*Clostridium*	Dejea [108]	2014	-	USA	T	CRC/CRA	Pyrosequencing
	Hibberd [105]	2017	-	USA	T	Colon cancer	16S rDNA Sequencing
	Zhang [109]	2018	2014–2015	China	**S**	CRC/A-CRA/BP	16S rDNA Sequencing
	Scanlan [110]	2008	-	Ireland	**S**	Colon cancer	16S rRNA Sequencing
	Allali [36]	2018	-	Morocco	**S**	CRC	16S rDNA Sequencing
	Kasai [88]	2015	2012–2013	Japan	**S**	CRC/CRA	T-RFLP/NGS
	Fukugaiti [111]	2015	-	Brazil	**S**	CRC	qPCR
	Ohigashi [112]	2013	2009–2010	Japan	**S**	CRC	qPCR
	Xie [113]	2017	2016	China	**S**	CRA/CRC/A-CRC	PCR
	Liang [101]	2016	-	China	**S**	CRC	duplex qPCR
*Collinsella*	Chen [94]	2012	-	China	**T**	CRC	16S rDNA Sequencing
*Desulfovibrio*	Hale [104]	2017	2001–2005	USA	**S**	CRA	16S rRNA Sequencing
	Chen [94]	2012	-	China	T	CRC	16S rDNA Sequencing
*Dialister*	Xu [96]	2017	-	China	T	CRC/CRA	NGS
	Zhang [109]	2018	2014–2015	China	**S**	CRC/A-CRA/BP	16S rDNA Sequencing
	Hibberd [105]	2017	-	USA	T	Colon cancer	16S rDNA Sequencing
*Dorea*	Peters [87]	2016	2012–2014	USA	**S**	CRC/CRA	Pyrosequencing
	Wu [91]	2013	-	China	**S**	CRC	16S rRNA Sequencing
	Hibberd [105]	2017	-	USA	**S**	Colon cancer	16S rRNA Sequencing
	Shen [114]	2010	-	USA	T	CRA	16S rDNA Sequencing
*Eggerthella*	Zhang [109]	2018	2014–2015	China	S	CRC/A-CRA/BP	16S rDNA Sequencing
*Enterococcus*	Chen [115]	2013	2010–2011	China	S	A-CRA	Pyrosequencing
	Wang [99]	2012	-	China	S	CRC	16S rDNA Sequencing
	Balamurugan [39]	2008	-	India	S	CRC	Real-time PCR
*Escherichia*	Feng [90]	2015	2010–2012	Austria	S	A-CRA/CRC	Metagenomic Shotgun Sequencing
	Xu [96]	2017	-	China	T	CRC/CRA	NGS
	Mori [116]	2018	2013–2015	Italy	S	CRC/CRA	16S rRNA Sequencing
	Wang [99]	2012	-	China	S	CRC	16S rRNA Sequencing
	Wu [91]	2013	-	China	S	CRC	16S rRNA Sequencing
	Goedert [117]	2015	-	USA	S	CRA	16S rRNA Sequencing
	Yoon [118]	2017	-	Korea	T	CRC/CRA	16S rDNA Sequencing
	Gao [100]	2015	-	China	T	CRC	16S rDNA Sequencing
	Mira-Pascual [89]	2015	-	Spain	T	CRC/CRA	qPCR
	Kohoutova [44]	2014	-	UK	T	CRC/CRA	PCR
	Bonnet [119]	2013	2007–2010	France	T	Colon cancer	PCR
	Swidsinski [120]	1998	-	Austria	T	CRC/CRA	PCR
*Eubacterium*	Chen [94]	2012	-	China	T	CRC	16S rDNA Sequencing
*Faecalibacterium*	Sze [121]	2017	-	USA	**S**	CRC/A-CRA/CRA	16S rDNA Sequencing
	Shen [114]	2010	-	USA	**T**	CRA	16S rRNA Sequencing
*Fastidiosipila*	Wu [91]	2013	-	China	S	CRC	16S rRNA Sequencing
*Fastidiosipila*	Wu [91]	2013	-	China	S	CRC	16S rRNA Sequencing
*Fusobacterium*	Vogtmann [92]	2016	1985–1987	USA	**S**	CRC	Whole-genome Shotgun Sequencing
	Feng [90]	2015	2010–2012	Austria	**S**	A-CRA/CRC	Metagenomic Shotgun Sequencing
	Yu [122]	2015	-	China	**S**	CRC	Metagenomic Sequencing
	Dejea [108]	2014	-	USA	**T**	CRC/CRA	Pyrosequencing
	Xu [96]	2017	-	China	**T**	CRC/CRA	NGS
	Deng [123]	2018	-	China	**S**	CRC	NGS
	Kasai [88]	2015	2012–2013	Japan	**S**	CRC/CRA	T-RFLP/ NGS
	Kostic [124]	2012	-	Spain	**T**	CRC	WGS/16S rDNA Sequencing/qPCR/FISH
	Allali [36]	2018	-	Morocco	**S**	CRC	16S rDNA Sequencing
	Zackular [125]	2014	-	Michigan	**S**	CRC/CRA	16S rDNA Sequencing
	Ahn [93]	2013	1985–1989	Washington	**S**	CRC	16S rDNA Sequencing
	Sinha [16]	2016	1985–1987	USA	**S**	CRC	16S rDNA Sequencing
	Flemer [98]	2015	-	Ireland	**S/T**	CRC/Polyps	16S rDNA Sequencing
	Flemer [126]	2017	-	Ireland	**S/T**	CRC/Polyps	16S rDNA Sequencing
	Zhang [109]	2018	2014–2015	China	**S**	CRC/A-CRA/BP	16S rDNA Sequencing
	Zeller [28]	2014	2004–2006	France/Germany	**S**	CRC/CRA	16S rDNA Sequencing
	Baxter [127]	2016	-	USA	**S**	CRC/CRA	16S rDNA Sequencing
	Gao [100]	2015	-	China	**T**	CRC	16S rDNA Sequencing
	Hibberd [105]	2017	-	USA	**T**	Colon cancer	16S rDNA Sequencing
	Chen [94]	2012	-	China	**T**	CRC	16S rDNA Sequencing
	Drewes [37]	2017	-	Malaysia	**T**	CRC	16S rDNA Sequencing
	Yoon [118]	2017	-	Korea	**T**	CRC/CRA	16S rDNA Sequencing
	Amitay [128]	2017	2005–2013	Germany	**S**	CRC/A-CRA/CRA	16S rDNA Sequencing/multiplex PCR
	Nakatsu [27]	2015	2011–2014	China	**T**	CRC/CRA	16S rRNA Sequencing/qPCR
	Wu [91]	2013	-	China	**S**	CRC	16S rDNA Sequencing/qPCR
	Russo [129]	2018	2015–2016	Italy	**S**	CRC	qPCR/16S rDNA Sequencing
	Liang [101]	2016	-	China	**S**	CRC	duplex qPCR
	Kostic [60]	2013	-	USA	**S**	CRC/CRA	qPCR
	Wong [130]	2016	-	China	**S**	CRC/A-CRA	qPCR
	Fukugaiti [111]	2015	-	Brazil	**S**	CRC	qPCR
	Eklof [131]	2017	2008–2013	Sweden	**S**	CRC	qPCR
	Mira-Pascual [89]	2015	-	Spain	**S**	CRC/CRA	qPCR
	Yu [122]	2015	-	China	**S**	CRC	qPCR
	Flanagan [132]	2014	2008–2010	Ireland	**S**	CRC	qPCR
	Repass [133]	2016	-	USA	**T**	CRC	qPCR
	Castellarin [134]	2012	-	Canada	**T**	CRC	qPCR
	Tahara [135]	2014	-	Japan	**T**	CRC	qPCR
	Ito [136]	2015	2001–2013	Japan	**T**	CRC	qPCR
	McCoy [137]	2013	-	USA	**T**	CRA	qPCR
	Suehiro [138]	2016	-	Japan	**S**	CRC/CRA/A-CRA	PCR
*Gemella*	Zhang [109]	2018	2014 - 2015	China	S	CRC/A-CRA/BP	16S rDNA Sequencing
	Baxter [127]	2016	-	USA	S	CRC/CRA	16S rDNA Sequencing
	Chen [94]	2012	-	China	T	CRC	16S rDNA Sequencing
	Nakatsu [27]	2015	2011–2014	China	T	CRC/CRA	16S rRNA Sequencing/qPCR
*Granulicatella*	Nakatsu [27]	2015	2011–2014	China	T	CRC/CRA	16S rRNA Sequencing/qPCR
*Heamophilus*	Kasai [88]	2015	2012–2013	Japan	S	CRC/CRA	T-RFLP/NGS
*Helicobacter*	Goedert [117]	2015	-	USA	S	CRA	16S rRNA Sequencing
	Sanapareddy [86]	2012	-	USA	T	CRA	16S rDNA Sequencing
*Klebsiella*	Wang [99]	2012	-	China	S	CRC	16S rDNA Sequencing
	Goedert [117]	2015	-	USA	S	CRA	16S rRNA Sequencing
	Chen [94]	2012	-	China	T	CRC	16S rDNA Sequencing
*Lactobacillus*	Xu [96]	2017	-	China	**T**	CRC/CRA	NGS
	Sanapareddy [86]	2012	-	USA	**T**	CRA	16S rDNA Sequencing
*Lactococcus*	Lu [139]	2016	2014	China	T	CRA	Pyrosequencing
	Sanapareddy [86]	2012	-	USA	T	CRA	16S rDNA Sequencing
	Gao [100]	2015	-	China	T	CRC	16S rDNA Sequencing
*Methanobrevibacter*	Hibberd [105] Mira-Pascual [89]	2017	-	USA	T	Colon cancer CRC/CRA	16S rDNA Sequencing
		2015	-	Spain	S		qPCR
*Methanosphaera*	Ai [107]	2017	2012	China	S	CRC/CRA	16S rRNA Sequencing
*Mogibacterium*	Xu [96]	2017	-	China	T	CRC/CRA	NGS
	Hale [104]	2017	2001–2005	USA	S	CRA	16S rRNA Sequencing
	Chen [94]	2012	-	China	T	CRC	16S rDNA Sequencing
*Morganella*	Goedert [117]	2015	-	USA	S	CRA	16S rRNA Sequencing
*Odoribacter*	Wu [91]	2013	-	China	S	CRC	16S rRNA Sequencing
*Oscillibacter*	Wu [91]	2013	-	China	S	CRC	16S rRNA Sequencing
	Flemer [98]	2015	-	Ireland	S/T	CRC/Polyps	16S rRNA Sequencing
*Oscillospira*	Deng [123]	2018	-	China	S	CRC	NGS
*Pantoea*	Goedert [117]	2015	-	USA	S	CRA	16S rRNA Sequencing
*Parabacteroides*	Feng [90]	2015	2010–2012	Austria	S	A-CRA/CRC	Metagenomic Shotgun Sequencing
*Parvimonas*	Feng [90]	2015	2010–2012	Austria	S	A-CRA/CRC	Metagenomic Shotgun Sequencing
	Yu [122]	2015	-	China	S	CRC	Metagenomic Sequencing
	Xu [96]	2017	-	China	T	CRC/CRA	NGS
	Zhang [109]	2018	2014–2015	China	S	CRC/A-CRA/BP	16S rDNA Sequencing
	Baxter [127]	2016	-	USA	S	CRC/CRA	16S rRNA Sequencing
	Flemer [98]	2015	-	Ireland	S/T	CRC/Polyps	16S rDNA Sequencing
	Flemer [126]	2017	-	Ireland	S/T	CRC/Polyps	16S rDNA Sequencing
	Sze [121]	2017	-	USA	S	CRC/A-CRA/CRA	16S rDNA Sequencing
	Drewes [37]	2017	-	Malaysia	T	CRC	16S rDNA Sequencing
	Nakatsu [27]	2015	2011–2014	China	T	CRC/CRA	16S rRNA Sequencing /qPCR
	Wong [130]	2016	-	China	S	CRC/A-CRA	qPCR
*Peptostreptococcus*	Yu [122]	2015	-	China	**S**	CRC	Metagenomic Sequencing
	Xu [96]	2017	-	China	T	CRC/CRA	NGS
	Zhang [109]	2018	2014–2015	China	**S**	CRC/A-CRA/BP	16S rDNA Sequencing
	Baxter [127]	2016	-	USA	**S**	CRC/CRA	16S rRNA Sequencing
	Zeller [28]	2014	2004–2006	France/Germany	**S**	CRC/CRA	16S rRNA Sequencing
	Flemer [98]	2015	-	Ireland	**S**/T	CRC/Polyps	16S rRNA Sequencing
	Flemer [126]	2017	-	Ireland	**S**/T	CRC/Polyps	16S rRNA Sequencing
	Hibberd [105]	2017	-	USA	**S**/T	Colon cancer	16S rRNA Sequencing
	Chen [94]	2012	-	China	T	CRC	16S rDNA Sequencing
	Drewes [37]	2017	-	Malaysia	T	CRC	16S rDNA Sequencing
	Gao [100]	2015	-	China	T	CRC	16S rDNA Sequencing
	Wang [99]	2012	-	China	**S**	CRC	16S rDNA Sequencing/qPCR
	Nakatsu [27]	2015	2011–2014	China	T	CRC/CRA	16S rRNA Sequencing/qPCR
*Phascolarctobacterium*	Weir [85]	2013	-	USA	S	CRC	16S rDNA Sequencing
	Wu [91]	2013	-	China	S	CRC	16S rDNA Sequencing
*Porphyromonas*	Vogtmann [92]	2016	1985–1987	USA	S	CRC	Whole-genome Shotgun Sequencing
	Sobhani [95]	2011	2004–2006	France	S	CRC	Pyrosequencing/qPCR
	Baxter [127]	2016	-	USA	S	CRC/CRA	16S rRNA Sequencing
	Allali [36]	2018	-	Morocco	S	CRC	16S rRNA Sequencing
	Zackular [125]	2014	-	Michigan	S	CRC/CRA	16S rRNA Sequencing
	Sze [121]	2017	-	USA	S	CRC/A-CRA/CRA	16S rRNA Sequencing
	Ahn [93]	2013	1985–1989	Washington	S	CRC	16S rRNA Sequencing
	Wang [99]	2012	-	China	S	CRC	16S rRNA Sequencing
	Sinha [16]	2016	1985–1987	USA	S	CRC	16S rRNA Sequencing
	Zhang [109]	2018	2014–2015	China	S	CRC/A-CRA/BP	16S rRNA Sequencing
	Zeller [28]	2014	2004–2006	France/Germany	S	CRC/CRA	16S rRNA Sequencing
	Flemer [98]	2015	-	Ireland	S/T	CRC/Polyps	16S rRNA Sequencing
	Chen [94]	2012	-	China	T	CRC	16S rDNA Sequencing
	Geng [140]	2014	-	China	T	CRC/CRA	16S rRNA Sequencing
*Prevotella*	Deng [123]	2018	-	China	**S**	CRC	NGS
	Kasai [88]	2015	2012–2013	Japan	**S**	CRC/CRA	T-RFLP/NGS
	Baxter [127]	2016	-	USA	**S**	CRC/CRA	16S rRNA Sequencing
	Flemer [126]	2017	-	Ireland	**S**/T	CRC/Polyps	16S rRNA Sequencing
	Gao [100]	2015	-	China	T	CRC	16S rDNA Sequencing
	Chen [94]	2012	-	China	T	CRC	16S rDNA Sequencing
	Mira-Pascual [89]	2015	-	Spain	T	CRC/CRA	16S rRNA Sequencing/qPCR
*Paraprevotella*	Chen [94]	2012	-	China	T	CRC	16S rDNA Sequencing
*Pseudomonas*	Lu [139]	2016	2014	China	**T**	CRA	Pyrosequencing
	Zackular [125]	2014	-	Michigan	S	CRC/CRA	16S rDNA Sequencing
	Goedert [117]	2015	-	USA	S	CRA	16S rRNA Sequencing
	Sanapareddy [86]	2012	-	USA	**T**	CRA	16S rDNA Sequencing
	Yoon [118]	2017	-	Korea	**T**	CRC/CRA	16S rDNA Sequencing
	Ohigashi [112]	2013	2009–2010	Japan	S	CRC	qPCR
*Pyramidobacter*	Yazici [106]	2017	2011–2012	USA	T	CRC	16S rRNA Sequencing
*Rhizobium*	Yoon [118]	2017	-	Korea	T	CRC/CRA	16S rDNA Sequencing
*Roseburia*	Flemer [98]	2015	-	Ireland	**S/T**	CRC/Polyps	16S rDNA Sequencing
	Liang [101]	2016	-	China	**S**	CRC	duplex qPCR
*Ruminococcus*	Dejea [108]	2014	-	USA	**T**	CRC/CRA	Pyrosequencing
	Allali [36]	2018	-	Morocco	**S**	CRC	16S rDNA Sequencing
	Zhang [109]	2018	2014–2015	China	**S**	CRC/A-CRA/BP CRC/Polyps	16S rDNA Sequencing
	Flemer [98]	2015	-	Ireland	**S/T**	CRA	16S rDNA Sequencing
	Shen [114]	2010	-	USA	**T**		16S rRNA Sequencing
*Salmonella*	Goedert [117]	2015	-	USA	S	CRA	16S rRNA Sequencing
*Selenomonas*	Allali [36]	2018	-	Morocco	**S**	CRC	16S rDNA Sequencing
	Hibberd [105]	2017	-	USA	T	Colon cancer	16S rDNA Sequencing
*Serratia*	Goedert [117]	2015	-	USA	S	CRA	16S rDNA Sequencing
*Slackia*	Chen [94]	2012	-	China	T	CRC	16S rDNA Sequencing
*Sphingomonas*	Richard [141]	2018	-	France	**T**	CAC^d^/CRC	qPCR/16S rRNA Sequencing
*Shigella*	Goedert [117]	2015	-	USA	S	CRA	16S rDNA Sequencing
	Mori [116]	2018	2013–2015	Italy	S	CRC/CRA	16S rRNA Sequencing
	Wang [99]	2012	-	China	S	CRC	16S rRNA Sequencing
	Wu [91]	2013	-	China	S	CRC	16S rRNA Sequencing
	Shen [114]	2010	-	USA	T	CRA	16S rRNA Sequencing
	Gao [100]	2015	-	China	T	CRC	16S rDNA Sequencing
	Mira-Pascual [89]	2015	-	Spain	T	CRC/CRA	qPCR
*Solobacterium*	Yu [122]	2015	-	China	S	CRC	Metagenomic Sequencing
	Zhang [109]	2018	2014–2015	China	S	CRC/A-CRA/BP	16S rDNA Sequencing
*Streptococcus*	Chen [115]	2013	2010–2011	China	**S**	A-CRA	Pyrosequencing
	Peters [87]	2016	2012–2014	USA	**S**	CRC/CRA	Pyrosequencing
	Kasai [88]	2015	2012–2013	Japan	**S**	CRC/CRA	T-RFLP/NGS
	Flemer [126]	2017	-	Ireland	**S/T**	CRC/Polyps	16S rDNA Sequencing
	Wang [99]	2012	-	China	**S**	CRC	16S rDNA Sequencing
	Gao [100]	2015	-	China	**T**	CRC	16S rDNA Sequencing
	Chen [94]	2012	-	China	**T**	CRC	16S rDNA Sequencing
	Geng [140]	2014	-	China	**T**	CRC/CRA	16S rRNA Sequencing
	Richard [141]	2018	-	France	**T**	CAC/CRC	qPCR/16S rRNA Sequencing
	Klein [82]	1977	-	Chicago	**S**	CRC	Culture
*Subdoligranulum*	Wu [91]	2013	-	China	S	CRC	16S rRNA Sequencing
*Sutterella*	Mori [116]	2018	2013–2015	Italy	S	CRC/CRA	16S rRNA Sequencing
	Hale [104]	2017	2001–2005	USA	S	CRA	16S rRNA Sequencing
*Trabulsiella*	Goedert [117]	2015	-	USA	S	CRA	16S rRNA Sequencing
*Veillonella*	Kasai [88]	2015	2012–2013	Japan	**S**	CRC/CRA	T-RFLP/NGS
	Geng [140]	2014	-	China	T	CRC/CRA	16S rRNA Sequencing
**Decreased Gut Bacteria**							
*Acidovorax*	Gao [100]	2015	-	China	**T**	CRC	16S rDNA Sequencing
*Acinetobacter*	Gao [100]	2015	-	China	T	CRC	16S rDNA Sequencing
*Alistipes*	Wang [99]	2012	-	China	**S**	CRC	16S rDNA Sequencing
	Nakatsu [27]	2015	2011–2014	China	T	CRC/CRA	16S rRNA Sequencing
*Anaerostipes*	Chen [94]	2012	-	China	T	CRC	16S rDNA Sequencing
*Atopobium*	Ohigashi [112]	2013	2009–2010	Japan	**S**	CRC	qPCR
*Bacteriodes*	Kostic [124]	2012	-	Spain	**T**	CRC	WGS
	Zackular [125]	2014	-	Michigan	**S**	CRC/CRA	16S rDNA Sequencing
	Wang [99]	2012	-	China	**S**	CRC	16S rDNA Sequencing
	Weir [85]	2013	-	USA	**S**	CRC	16S rDNA Sequencing
	Allali [36]	2018	-	Morocco	**S**	CRC	16S rDNA Sequencing
	Shen [114]	2010	-	USA	**T**	CRA	16S rDNA Sequencing
	Nakatsu [27]	2015	2011–2014	China	**T**	CRC/CRA	16S rDNA Sequencing
	Ohigashi [112]	2013	2009–2010	Japan	**S**	CRC	qPCR
*Bacillus*	Lu [139]	2016	2014	China	T	CRA	Pyrosequencing
	Mira-Pascual [89]	2015	-	Spain	T	CRC/CRA	qPCR
*Bifidobacterium*	Feng [90]	2015	2010–2012	Austria	S	A-CRA/CRC	Metagenomic Shotgun Sequencing
	Chen [94]	2012	-	China	**T**	CRC	16S rDNA Sequencing
	Mira-Pascual [89]	2015	-	Spain	S	CRC/CRA	qPCR
	Ohigashi [112]	2013	2009–2010	Japan	S	CRC	qPCR
	Yusuf [38]	2016	-	Indonesia	S	CRC	DGGE
*Blautia*	Xu [96]	2017	-	China	**T**	CRC/CRA	NGS
	Chen [94]	2012	2011–2014	China	**T**	CRC	16S rDNA Sequencing
	Nakatsu [27]	2015	-	China	**T**	CRC/CRA	16S rDNA Sequencing
	Gao [100]	2015	-	China	**T**	CRC	16S rDNA Sequencing
*Buttiauxella*	Gao [100]	2015	-	China	T	CRC	16S rDNA Sequencing
*Caulobacter*	Gao [100]	2015	-	China	T	CRC	16S rRNA Sequencing
*Collinsella*	Nakatsu [27]	2015	2011–2014	China	**T**	CRC/CRA	16S rRNA Sequencing
*Clostridium*	Chen [115]	2013	2010–2011	China	**S**	A-CRA	Pyrosequencing
	Zackular [125]	2014	-	Michigan	**S**	CRC/CRA	16S rDNA Sequencing
	Ohigashi [112]	2013	2009–2010	Japan	**S**	CRC	qPCR
*Coprococcus*	Vogtmann [92]	2016	1985–1987	USA	S	CRC	Whole-genome Shotgun Sequencing
	Ahn [93]	2013	1985–1989	USA	S	CRC	16S rDNA Sequencing
	Zhang [109]	2018	2014–2015	China	S	CRC/A-CRA/BP	16S rDNA Sequencing
	Flemer [98]	2015	-	Ireland	S/T	CRC/Polyps	16S rDNA Sequencing
	Shen [114]	2010	-	USA	T	CRA	16S rRNA Sequencing
*Desulfovibrio*	Scanlan [142]	2009	-	UK	**S**	CRC	qPCR
*Dialister*	Weir [85]	2013	-	USA	**S**	CRC	16S rDNA Sequencing
*Dorea*	Weir [85]	2013	-	USA	**S**	CRC	16S rDNA Sequencing
*Enterococcus*	Lu [139]	2016	2014	China	T	CRA	Pyrosequencing
*Epilithonimonas*	Gao [100]	2015	-	China	T	CRC	16S rDNA Sequencing
*Eubacterium*	Yu [122]	2015	-	China	S	CRC	Metagenomic Sequencing
	Chen [115]	2013	2010–2011	China	S	A-CRA	Pyrosequencing
	Kasai [88]	2015	2012–2013	Japan	S	CRC/CRA	T-RFLP/NGS
	Wang [99]	2012	-	China	S	CRC	16S rDNA Sequencing
	Zhang [109]	2018	2014–2015	China	S	CRC/A-CRA/BP	16S rDNA Sequencing
	Balamurugan [39]	2008	-	India	S	CRC	Real-time PCR
	Vargo [83]	1980	-	USA	S	Colon cancer	Culture
*Faecalibacterium*	Xu [96]	2017	-	China	**T**	CRC/CRA	NGS
	Wu [91]	2013	-	China	**S**	CRC	16S rRNA Sequencing
	Chen [94]	2012	-	China	**T**	CRC	16S rDNA Sequencing
	Nakatsu [27]	2015	2011–2014	China	**T**	CRC/CRA	16S rRNA Sequencing
	Balamurugan [39]	2008	-	India	**S**	CRC	Real-time PCR
	Mira-Pascual [89]	2015	-	Spain	**T**	CRC/CRA	qPCR
	Lopez-Siles [143]	2016	-	Spain	**T**	CRC	qPCR
*Fusicatenibacter*	Zhang [109]	2018	2014–2015	China	S	CRC/A-CRA/BP	16S rDNA Sequencing
*Flavobacterium*	Gao [100]	2015	-	China	T	CRC	16S rDNA Sequencing
*Fusobacterium*	Shen [114]	2010	-	USA	**T**	CRA	16S rRNA Sequencing
	Richard [141]	2018	-	France	**T**	CAC/CRC	qPCR/16S rRNA Sequencing
	Vargo [83]	1980	-	USA	**S**	Colon cancer	Culture
*Haemophilus*	Hale [104]	2017	2001–2005	USA	S	CRA	16S rRNA Sequencing
*Janthinobacterium*	Gao [100]	2015	-	China	T	CRC	16S rDNA Sequencing
*Lachnobacterium*	Weir [85]	2013	-	USA	S	CRC	16S rDNA Sequencing
*Lachnospira*	Weir [85]	2013	-	USA	S	CRC	16S rDNA Sequencing
	Chen [94]	2012	-	China	T	CRC	16S rDNA Sequencing
*Lactobacillus*	Feng [90]	2015	2010–2012	Austria	S	A-CRA/CRC	Metagenomic Shotgun Sequencing
	Chen [94]	2012	-	China	**T**	CRC	16S rDNA Sequencing
*Megamonas*	Weir [85]	2013	-	USA	S	CRC	16S rDNA Sequencing
*Megasphaera*	Ahn [93]	2013	1985–1989	Washington	S	CRC	16S rDNA Sequencing
*Parasutterella*	Wang [99]	2012	-	China	S	CRC	16S rDNA Sequencing
*Pedobacter*	Gao [100]	2015	-	China	T	CRC	16S rDNA Sequencing
*Propionibacterium*	Gao [100]	2015	-	China	T	CRC	16S rDNA Sequencing
*Peptostreptococcus*	Ahn [93]	2013	1985–1989	Washington	**S**	CRC	16S rDNA Sequencing
*Prevotella*	Weir [85]	2013	-	USA	**S**	CRC	16S rDNA Sequencing
*Pseudobutyrivibrio*	Weir [85]	2013	-	USA	S	CRC	16S rDNA Sequencing
	Chen [94]	2012	-	China	T	CRC	16S rDNA Sequencing
*Pseudomonas*	Gao [100]	2015	-	China	**T**	CRC	16S rDNA Sequencing
*Psychrobacter*	Gao [100]	2015	-	China	T	CRC	16S rDNA Sequencing
*Rahnella*	Gao [100]	2015	-	China	T	CRC	16S rDNA Sequencing
*Roseburia*	Chen [115]	2013	2010–2011	China	**S**	A-CRA	Pyrosequencing
	Wang [99]	2012	-	China	**S**	CRC	16S rDNA Sequencing
	Wu [91]	2013	-	China	**S**	CRC	16S rRNA Sequencing
	Hibberd [105]	2017	-	USA	**S**	Colon cancer	16S rRNA Sequencing
	Zhang [109]	2018	2014–2015	China	**S**	CRC/A-CRA/BP	16S rRNA Sequencing
	Chen [94]	2012	-	China	**T**	CRC	16S rDNA Sequencing
*Ruminococcus*	Feng [90]	2015	2010–2012	Austria	**S**	A-CRA/CRC	Metagenomic Shotgun Sequencing
	Ahn [93]	2013	1985–1989	Washington	**S**	CRC	16S rDNA Sequencing
	Weir [85]	2013	-	USA	**S**	CRC	16S rDNA Sequencing
	Zhang [109]	2018	2014–2015	China	**S**	CRC/A-CRA/BP	16S rDNA Sequencing
	Richard [141]	2018	-	France	**T**	CAC/CRC	qPCR/16S rRNA Sequencing
*Selenomonas*	Ahn [93]	2013	1985–1989	Washington	**S**	CRC	16S rDNA Sequencing
*Slackia*	Kasai [88]	2015	2012–2013	Japan	S	CRC/CRA	T-RFLP/NGS
*Solibacillus*	Lu [139]	2016	2014	China	T	CRA	Pyrosequencing
*Sphingobacterium*	Gao [100]	2015	-	China	T	CRC	16S rDNA Sequencing
*Sphingomonas*	Gao [100]	2015	-	China	**T**	CRC	16S rDNA Sequencing
*Staphylococcus*	Ohigashi [112]	2013	2009–2010	Japan	S	CRC	qPCR
	Mira-Pascual [89]	2015	-	Spain	T	CRC/CRA	qPCR
*Streptococcus*	Feng [90]	2015	2010–2012	Austria	**S**	A-CRA/CRC	Metagenomic Shotgun Sequencing
	Hale [104]	2017	2001–2005	USA	**S**	CRA	16S rRNA Sequencing
	Zhang [109]	2018	2014–2015	China	**S**	CRC/A-CRA/BP	16S rDNA Sequencing
	Hibberd [105]	2017	-	USA	**T**	Colon cancer	16S rDNA Sequencing
	Sanapareddy [86]	2012	-	USA	**T**	CRA	16S rDNA Sequencing
*Stenotrophomonas*	Gao [100]	2015	-	China	T	CRC	16S rDNA Sequencing
*Sutterella*	Nakatsu [27]	2015	2011–2014	China	T	CRC/CRA	16S rRNA Sequencing/qPCR
*Veillonella*	Hale [104]	2017	2001–2005	USA	**S**	CRA	16S rRNA Sequencing

^a^ Stool/Tissue. ^b^ Advanced colorectal adenoma. ^c^ Benign polyp. ^d^ Colitis-associated cancer. Gut bacteria isolated from stool or tissue samples with both increased and decreased evidence are presented in bold.

**Table 2:** Evidence of the microbial-derived metabolites in CRC.

Metabolite	Chemical Class	Bacterial Source	Bacterial Level in CRC ^a^	Reference
Benzoic Acid	Benzenoid (Benzene)	*Serratia*	+	[15,16,171]
Hippuric Acid(Benzamidoacetic Acid)	Benzenoid (Benzene)	*Clostridium* *Eubacterium* *Ruminococcus* *Faecalibacterium*	± ± ± ±	[171]
Hydroxybenzoic Acid	Benzenoid (Benzene)	*Arthrobacter* *Bifidobacterium* *Microbulbifer* *Escherichia* *Eubacterium* *Corynebacterium* *Clostridium*	* ± * ± ± * ±	[16,171]
Syringic Acid	Benzenoid (Benzene)	*Bifidobacterium*	±	[171]
3-Hydroxyphenylacetic Acid	Benzenoid (Phenol)	*Klebsiella* *Clostridium*	+ ±	[15,171]
4-Hydroxyphenylacetic Acid	Benzenoid (Phenol)	*Pseudomonas* *Klebsiella* *Acinetobacter* *Clostridium*	± + - ±	[15,16,171]
p-Cresol	Benzenoid (Phenol)	*Bacteriodes* *Bifidobacterium* *Enterobacter* *Lactobacillus* *Clostridium*	± ± * ± ±	[15]
Allantoin	Organoheterocyclic Compound (Azole)	*Bacillus* *Streptomyces*	- *	[171]
N-Acetylputrescine	Organic Acid (Organic Carboximidic Acid)	*Corynebacterium*	*	[15,16,171]
5-Aminopentanoic Acid	Organic Acid (Organic Carboximidic Acid)	*Corynebacterium*	*	[15,16,171]
Acetic Acid	Organic Acid (Organic Carboximidic Acid)	*Acinetobacter* *Bacteriodes* *Bifidobacterium* *Enterobacter* *Prevotella* *Ruminococcus* *Streptococcus* *Staphylococcus* *Pseudomonas* *Proteus* *Klebsiella* *Escherichia* *Enterococcus* *Citrobacter* *Akkermansia*	- ± ± * ± ± ± - ± * + ± ± + +	[85,172,173,174,175,176]
Gamma-Aminobutyric Acid (GABA)	Organic Acid (Organic Carboximidic Acid)	*Bifidobacterium* *Lactobacillus*	± ±	[15,16,171]
Glutaric Acid	Organic Acid (Organic Carboximidic Acid)	*Escherichia*	±	[15,16,171]
Succinic Acid	Organic Acid (Organic Carboximidic Acid)	*Acinetobacter* *Enterobacter* *Corynebacterium* *Basfia* *Pseudomonas* *Proteus* *Mannheimia* *Klebsiella* *Escherichia* *Enterococcus* *Citrobacter* *Anaerobiospirillum* *Actinobacillus*	- * * * ± * * + ± ± + * *	[15,16,171,174]
5-Keto-D-gluconate	Organic Acid (Organic Hydroxy Acid)	*Gluconobacter*	*	[15,171]
Hydroxypropionic Acid	Organic Acid (Organic Hydroxy Acid)	*Escherichia* *Klebsiella*	± +	[15,16,171]
Lactic Acid	Organic Acid (Organic Hydroxy Acid)	*Acinetobacter* *Enterobacter* *Corynebacterium* *Bacillus* *Streptococcus* *Staphylococcus* *Pseudomonas* *Proteus* *Klebsiella* *Escherichia* *Enterococcus* *Citrobacter*	- * * - ± - ± * + ± ± +	[15,16,171,174,176]
Hydroxyacetic Acid(Glycolic Acid)	Organic Acid (Organic Hydroxy Acid)	*Alcaligenes* *Acetobacter* *Rhodococcus* *Pseudomonas* *Leptospirillum* *Gluconobacter* *Escherichia* *Acidithiobacillus* *Corynebacterium*	* * * ± * * ± * *	[15,16,171]
Pyruvic Acid	Organic Acid (Organic Keto Acid)	*Corynebacterium* *Escherichia*	* ±	[16,171]
Oxoglutaric Acid(Ketoglutaric Acid)	Organic Acid (Organic Keto Acid)	*Corynebacterium*	*	[15]
p-Cresol sulfate	Organic Acid (Organic Sulfuric Acid)	*Clostridium* *Lactobacillus* *Enterobacter* *Bifidobacterium*	± ± * ±	[15,16,171]
Cadaverine	Organonitrogen Compound (Amine)	*Corynebacterium*	*	[15,16,171]
Putrescine	Organonitrogen Compound (Amine)	*Enterobacter* *Cronobacter* *Citrobacter* *Corynebacterium*	* * + *	[15,16,171]
2,3-Butanediol	Organooxygen Compound (Alcohol)	*Serratia* *Klebsiella* *Bacillus* *Enterobacter*	+ + - *	[15]
D-Arabinose	Organooxygen Compound (Carbohydrate)	*Streptococcus* *Pediococcus* *Lactococcus* *Lactobacillus* *Geobacillus* *Escherichia* *Enterococcus* *Enterobacter* *Clostridium* *Alicyclobacillus* *Bifidobacterium*	± * + ± * ± ± * ± * ±	[15]
Mannitol	Organooxygen Compound (Carbohydrate)	*Clostridium* *Streptococcus* *Leuconostoc* *Zymomonas* *Torulaspora* *Rhodobacter* *Pseudomonas* *Lactococcus* *Gluconobacter* *Lactobacillus*	± ± * * * * ± + * ±	[171]
Ribulose	Organooxygen Compound (Carbohydrate)	*Acetobacter* *Gluconobacter*	* *	[15]
Tartaric Acid	Organooxygen Compound (Carbohydrate)	*Agrobacterium* *Nocardia* *Rhizobium*	* * +	[171]
Indoleacetic Acid	Organoheterocyclic Compound (Indole)	*Bradyrhizobium* *Rhizobium* *Pseudomonas* *Pantoea* *Enterobacter* *Clostridium* *Bacillus* *Agrobacterium* *Azospirillum*	* + ± + * ± - * *	[15,16,171]
5-Hydroxytryptamine(Serotonin)	Indole	*Enterococcus* *Streptococcus* *Escherichia*	± ± ±	[15]
Tryptamine	Indole	*Ruminococcus* *Clostridium*	± ±	[15,171]
Ferulic Acid	Phenylpropanoid Polyketide (Phenylpropanoic Acid)	*Pseudomonas*	±	[15,16,171]
Desaminotyrosine (4-Hydroxyphenylpropionic Acid)	Phenylpropanoid Polyketide (Phenylpropanoic Acid)	*Klebsiella* *Staphylococcus* *Pseudomonas* *Lactobacillus* *Eubacterium* *Enterococcus* *Clostridium* *Bifidobacterium* *Acinetobacter* *Bacteriodes*	+ - ± ± ± ± ± ± - ±	[15,16,171]
Hydrocinnamic Acid	Phenylpropanoid Polyketide (Phenylpropanoic Acid)	*Clostridium* *Eubacterium*	± ±	[15,16,171]
Hydroxyphenyllactic Acid	Phenylpropanoid Polyketide (Phenylpropanoic Acid)	*Clostridium* *Bifidobacterium* *Staphylococcus* *Pseudomonas* *Lactobacillus* *Klebsiella* *Eubacterium* *Escherichia* *Enterococcus* *Acinetobacter* *Bacteriodes*	± ± - ± ± + ± ± ± - ±	[15,171]
Phenyllactic Acid	Phenylpropanoid Polyketide (Phenylpropanoic Acid)	*Clostridium Klebsiella* *Staphylococcus* *Pseudomonas* *Lactobacillus* *Eubacterium* *Escherichia* *Enterococcus* *Bifidobacterium* *Acinetobacter* *Bacteriodes*	± + - ± ± ± ± ± ± - ±	[15]
6-Hydroxynicotinic Acid	Organoheterocyclic Compound (Pyridine)	*Serratia* *Achromobacter* *Pseudomonas*	+ * ±	[15,16,171]
Butyric Acid	Lipid (Fatty Acyl)	*Anaerostipes* *Eubacterium* *Roseburia* *Faecalibacterium* *Coprococcus*	- ± ± ± -	[85,174,176]
Coprosterol	Steroid (Cholesterol)	*Lactobacillus*	±	[15]
Glycocholic Acid	Steroid (Bile Acid)	*Bacteriodes* *Bifidobacterium* *Clostridium* *Lactobacillus*	± ± ± ±	[15,16,171]

^a^ The bacterial relative abundance in CRC based on reported data in Table 1. Increase (+), Decrease (-), both increase and decrease (±), Not available (*).
